# 3D geodynamic-geomorphologic modelling of deformation and exhumation at curved plate boundaries: Implications for the southern Alaskan plate corner

**DOI:** 10.1038/s41598-022-17644-8

**Published:** 2022-08-22

**Authors:** Alexander Koptev, Matthias Nettesheim, Sarah Falkowski, Todd A. Ehlers

**Affiliations:** 1 Department of Geosciences, University of Tübingen, Tübingen, Germany; 2 GFZ German Research Centre for Geosciences, Potsdam, Germany

**Keywords:** Geodynamics, Geomorphology, Tectonics

## Abstract

Plate corners with extreme exhumation rates are important because they offer a perspective for understanding the interactions between tectonics and surface processes. The southern Alaskan margin with its curved convergent plate boundary and associated zones of localized uplift is a prime location to study active orogeny. Here, we present the results of fully-coupled thermo-mechanical (geodynamic) and geomorphologic numerical modelling, the design of which captures the key features of the studied area: subduction of oceanic lithosphere (Pacific plate) is adjacent to a pronounced asymmetric indenter dipping at a shallow angle (Yakutat microplate), which in turn is bounded to the east by a dextral strike-slip shear zone (Fairweather fault). The resulting first-order deformation/rock uplift patterns show strong similarities with observations. In particular, relatively young thermochronological ages are reproduced along the plate-bounding (Fairweather) transform fault and in the area of its transition to convergence (the St. Elias syntaxis). The focused exhumation of the Chugach Core also finds its equivalent in model predicted zones of high rock uplift rates in an isolated region above the indenter. From these results, we suggest that the general exhumation patterns observed in southern Alaska are controlled by mutually reinforcing effects of tectonic deformation and surface erosion processes.

## Introduction

Recent technical and conceptual advances in thermo-mechanical^1–6^ and landscape evolution^7–10^ numerical modelling have expanded the ability to investigate the combined effects of geodynamic processes and geomorphic conditions^11–14^ in different tectonic settings including plate corners. Plate corners are curved segments of convergent plate boundaries and give rise to diverse and complex tectonic and geomorphic processes. Deformation partitioning in such kinematic transition zones exhibits considerable spatial and temporal variations depending on plate geometry^15^, rheological properties of the overriding plate^16^, and intensity of surface erosion^17^. Spatially localized lithospheric deformation and focused rapid rock uplift (> 5 mm yr^−1^), which is commonly detected at orogen syntaxes^18–20^, have long attracted the attention of geoscientists.

According to the “tectonic aneurysm” hypothesis^21–23^, rapid exhumation of rocks is promoted by vigorous fluvial or glacial incision that erodes the cold and strong uppermost crust, thus leading to temperature-dependent crustal weakening and increased surface uplift. This surface uplift further intensifies erosional processes and exhumation. A prominent counter-argument to the “tectonic aneurysm” hypothesis emphasizes the crucial role of the geometry of the subducting slab at plate corners for initiating localized deformation and exhumation in orogens^15^. The coupled thermo-mechanical/landscape evolution modelling^17,24^ has reconciled these end-member views on deep and fast exhumation at plate corners showing that the highest rock uplift rates are concentrated in areas where a large erosion potential spatially coincides with strong tectonic forces.

Despite progress in numerical modelling of orogen syntaxes, previous studies were limited to a symmetrical indenter bulge^15–17,24^ introduced to mimic slab bending at plate corners^25–27^. Such generic model setups may only be applicable to plate boundaries with moderate and/or symmetric slab curvature, such as the South American^28,29^ or Cascadia^30,31^ subduction zones. Plate corners where convergence transitions to strike-slip motion, as on the southern Alaskan margin^18,32–34^, require a more specific model configuration that includes asymmetric structural features linked with the lateral change from subduction/collision to transform tectonics.

In this study, we present results from 3D coupled geodynamic-geomorphologic model simulations that include all characteristic geodynamic elements of the asymmetric southern Alaskan plate corner. Our main objective is to quantify the consequences of the implementation of realistic fluvial erosion and the relationship between the different components of convergence—lower (Pacific) plate subduction and upper (North American) plate advance—for the deformation distribution in the overriding continental lithosphere and for the resulting spatial location of the areas of focused and rapid rock exhumation reported in southern Alaska.

## Southern Alaskan plate corner: geodynamic background, tectonic history, and geomorphic signatures

**Figure 1:**
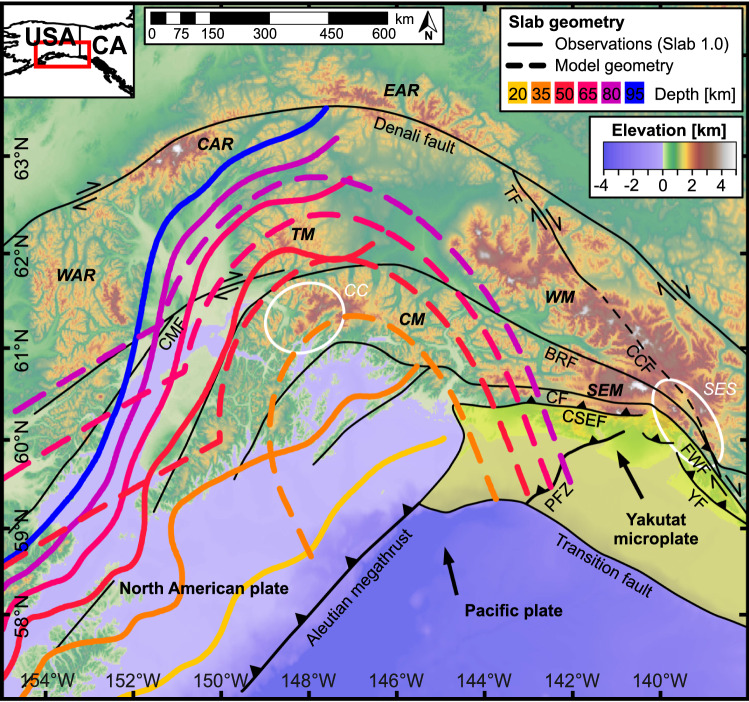
Tectonic overview of the southern Alaskan plate corner. Major faults^35,36^ are shown as black lines: BRF—Border Ranges fault, CCF—Connector fault, CF—Contact fault, CMF—Castle Mountain fault, CSEF—Chugach-St. Elias fault, FWF—Fairweather fault, PFZ—Pamplona fault zone, TF—Totschunda fault, YF—Yakutat fault. Mountain ranges are marked with bold, italic letters: CAR—Central Alaska Range, CM—Chugach Mountains, EAR—Eastern Alaska Range, SEM—St. Elias Mountains, TM—Talkeetna Mountains, WAR—Western Alaska Range, WM—Wrangell Mountains. White ellipses: CC—Chugach Core, SES—St. Elias syntaxis. Two bold arrows indicate the direction of Pacific and Yakutat plate motion. The solid colored lines indicate the depth contours corresponding to the observed configuration of the subducting interface from the global 3D model of subduction zones geometry (Slab 1.0; ref.^26^). The dashed lines show the approximated geometry of the downgoing plate used in our models.

The geodynamics of the southern Alaskan plate corner is determined by the ongoing subduction of the Pacific plate and the Yakutat microplate beneath the overriding North American plate (Fig. 1). The tectonic history of this region is associated with the accretion of several allochthonous terranes since the Paleozoic^35,36^. Subduction of the youngest in the sequence, the Yakutat microplate^37^, a wedge-shaped, 10–32 km thick oceanic plateau, established in the late Eocene^38–40^ and was followed by an oblique collision that began in Miocene time^41–43^. The resulting contrast in the geometry of the subduction interface at the Alaskan plate corner is quite remarkable^38,44,45^: to the west, the steeply dipping Pacific plate subducts along the Aleutian Megathrust, while at the plate corner apex, it transitions into a prominent indenter structure (Yakutat microplate) going downward at a shallow angle^26,46^ (see inferred plate contours in Fig. 1). Further east, the asymmetric body of the Yakutat microplate is bounded by the transpressional Fairweather fault.

The Alaskan orocline includes several mountain ranges with rugged topography and summit heights exceeding 5 km. Numerous studies have contributed to unravelling the topographic response to the complex geodynamic setting in the southern Alaskan plate corner^42,47–49^. Among others, studies from the St. Elias Mountains and the northern Fairweather Range have found evidence of rapid exhumation starting after ~ 10 Ma with the highest rates exceeding 5 mm yr^-1^ after ~ 5 Ma in the St. Elias syntaxis area (Fig. 1; refs.^43,50–52^). The shift in the location of rapid exhumation to the south after ~ 5 Ma and again further south at 3–2 Ma (refs.^52,53^) could result from a change in Pacific-North American plate motions and enhanced transpression at ~ 8 Ma (ref.^54^). Alternatively, it could be a consequence of a progressive collision of the buoyant and asymmetric wedge-shaped (thicker to the east) Yakutat microplate^38,52,53^. In addition, topographic elevations have reached levels sufficient to support glaciers since 6–5 Ma, which has altered patterns and intensity of erosion^53^. Exhumation along the Fairweather fault occurred since the Late Eocene, but the most rapid rates have been suggested for the past 3–2 Myr (2–6 mm yr^−1^and locally 5–10 mm yr^−1^; refs.^55,56^). In addition, the central Chugach Mountains are another site of focused exhumation (Chugach Core in Fig. 1) with an increase in rates after ~ 5 Ma, although rock uplift there is much slower (maximum average rates of ∼0.7 mm yr^−1^ over the past ~ 10 Myr; refs.^33,57^). Finally, the Alaska Range along the central and western segments of the Denali fault has also been characterized by spatially and temporally variable rapid exhumation since ~ 24 Ma. Thermochronometer-derived long-term exhumation rates (since ~ 24 Ma) for the Eastern Alaska Range were reported to be ~ 0.9 mm yr^−1^ (ref.^58^) and shorter-term rates (since ~ 6 Ma) for the Denali area in the Central Alaska Range were determined to be ~ 1 mm yr^−1^ (ref.^59^).

## Modelling approach and results

**Figure 2:**
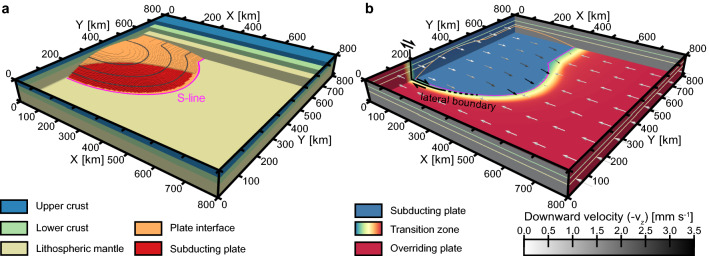
3D view of the model setup. (**a**) Material layout: a vertically layered overriding plate with upper crust, lower crust, and lithospheric mantle is underlain by a subducting plate with a cylindrical slab and a convex-upward-shaped indenter in the back and middle part of the model box, respectively. To emulate the asymmetric geometry of the southern Alaskan plate corner, the indenter bulge curves strongly towards the front part of the model, where the subducting slab is absent, in contrast to previous studies that assumed a symmetric model configuration^15–17,24^. The shape of the indenter is highlighted by gray isolines. The overriding and subducting plates are separated by a rheologically weak interface layer. To better illustrate the internal structure of the model, the plate interface is not shown in the lower part of the indenter. The S-line represents the intersection of the downgoing plate with the bottom of the model domain. (**b**) Velocity boundary conditions: the horizontal velocities applied to the left and right sides of the model box cause the overriding plate to move horizontally in the negative X-direction (red color), while the subducting plate moves in the opposite course (blue color). The inflow of lower plate material is mass-balanced by a vertical outflow through the bottom surface, which gradually increases from the left side of the model towards the S-line (see the downward component of the velocity vectors shown in shades of gray), corresponding to a rotational motion of the subducting slab. The resulting relative motion of the overriding and subducting plates along the transitional zone of the weak interface (gradual color transitions) includes convergence (corresponding to the Aleutian Megathrust subduction zone) and dextral transform (the strike-slip Fairweather fault) at the principal (X-perpendicular) and lateral (X-parallel) boundaries of the subducting plate, respectively. See “Methods” for more details on the model setup.

The setup of 3D experiments presented here considers the major features of the southern Alaskan plate corner (Fig. 1). In contrast to previous studies that assume subduction of a straight wedge bounded on its side by a strike-slip fault^60^ or a symmetrical indenter bulge^15–17,24^, we introduce a more complex model configuration (Fig. 2). The cylindrical slab in the rear part of the model domain (corresponding to a steeply dipping Pacific plate) is adjacent to the ellipsoid body of the indenter (the more shallowly dipping Yakutat microplate), which is located in the central segment of the model. Consistent with the observed configuration of the Yakutat microplate, the central indenter is tightly curved to the front portion of the model box, which, unlike the rear, has no subducting plate. As a result, the S-line (i.e., the intersection of the subducting slab and indenter with the bottom of the model box as an analogy to the S-point definition^61^) is characterized by a highly asymmetric shape (see Fig. 2a). The velocity boundary conditions follow the model geometry dividing the model domain into two regions, which correspond to the upper (overriding) and lower (subducting) plates moving in opposite (convergent) directions (see Fig. 2b). The resulting horizontal motion along the lateral boundary of the subducting slab implies the presence of a dextral strike-slip shear zone (Fairweather fault) that wraps around the indenter and gradually transitions into a thrust fault (Aleutian Megathrust subduction zone).

This model configuration, together with velocity boundary conditions, was implemented in 3D geodynamic-geomorphologic numerical simulations using the thermo-mechanical code DOUAR^2,62^, which is fully coupled with the landscape evolution code FastScape^7^ (see “Methods” for more details).

The various experiments presented in our study involve different combinations of two control parameters: (1) the type of surface erosion (total or fluvial), and (2) the proportion of upper plate advance to total shortening (half or none). In the total (or flat) erosion scenario, all topography resulting from geodynamic/tectonic processes modelled by DOUAR is immediately eroded down to the original base level. The more realistic coupled geodynamic-geomorphologic scenarios (short: fluvial) use the FastScape algorithms to model fluvial erosion and hillslope diffusion. In the models without the upper plate advance component, convergence is completely accommodated by subduction of the downgoing plate, while the half upper plate advance scenario assumes an equal partitioning of boundary velocities.

**Figure 3:**
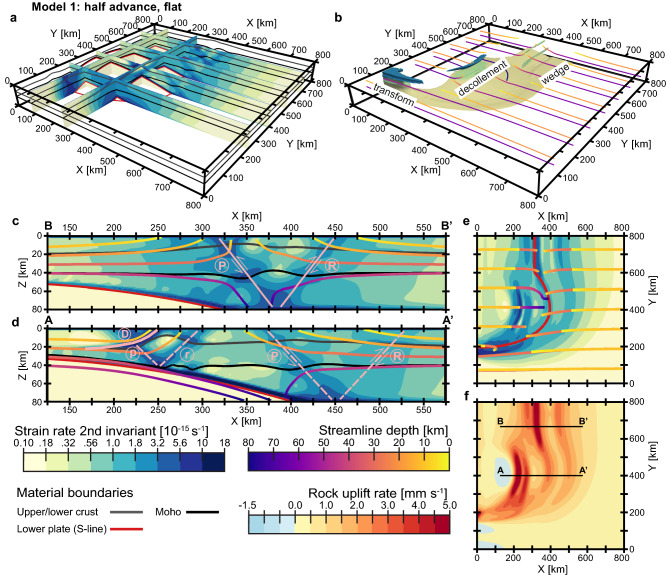
Overall view of model 1 (total erosion; half upper plate advance) after 6 Myr modelling time. (**a**–**e**) Second invariant of strain rate shown in 3D view (panels “a” and “b”), vertical cross-sections parallel to the X-axis (panels “c” and “d”), and plan view (panel “e”). (**f**) Top view of rock uplift rates at the surface. Panels “b” to “e” combine the strain rates with the motion streamlines. Panel “b” denotes the part of the model domain characterized by high strain rates (> 5·10^−15^ s^−1^), with colors corresponding to material layers as in Fig. 2. In panels “c” and “d”, the pro- and retro- shear zones are marked with “P” and “R” (solid pink lines in panel “c”) and “p” and “r” (dashed pink lines in panel “d”) for structures on lithospheric and crustal scales, respectively. The shallow retro-decollement is labelled by “D” (solid pink line in panel “d”). The shadowy continuations of the lithospheric-scale (pro- and retro-) shear zones into the central part of the model are interpreted by dashed pink lines in panel “d”. The positions of the vertical cross-sections shown in panels “c” and “d” are indicated by the black lines in panel “f”. The map view in panel “e” corresponds to strain rates at 5 km depth, while the particle trajectories (streamlines) shown there originate at 10 and 30 km depth. In the vertical cross-sections, the corresponding relatively shallow motion streamlines show exhumation of upper and lower crustal material, that increases from both sides towards the model center and accelerates dramatically at the main shear zones (panels “c” and “d”). In the horizontal plane, these flow lines generally demonstrate a nearly parallel influx of material, while in the front portion of the model they pass around the indenter, deflecting slightly in the negative Y-direction (panel “e”).

The first experiment (model 1; Fig. 3) is characterized by total (flat) erosion and half upper plate advance. Naturally, high values of the resulting strain rates are located at the interface between the subducting and upper plates, particularly in the lower half of the model (Fig. 3a–b). Within the lithosphere of the overriding plate, the bands of localized deformation form three main structures (Fig. 3b): (1) a lithospheric-scale wedge located at the rear part of the model and confined between two oppositely dipping (pro- and retro-) thrust-sense shear zones rooting at the model bottom near its intersection with the subducting plate (Fig. 3c); (2) the shallowly dipping decollement situated in the center of the model directly above the indenter and accompanied by crustal-scale shear zones that nucleate where the overriding plate’s Moho intersects with the subducting plate (Fig. 3d); and (3) the transform fault at the tightly curved edge of the indenter (Fig. 3e). It is noteworthy that the steeply dipping shear zones that dominate the rear part of the model attenuate markedly towards the middle of the model, where most of the deformation is accommodated by the shallowly dipping decollement (Fig. 3d). Nevertheless, their shadowy continuations remain discernible in the strain rate field, especially the pro-shear zone which is also more developed and pronounced in the rear segment of the model domain (Fig. 3c). Consistent with this deformation pattern, the highest rock uplift rates (> 5 mm yr^−1^; Fig. 3f) are focused in the hanging wall of the thrust-sense decollement (Fig. 3d) and along the pro-branch of the orogenic wedge (Fig. 3c). In the strike-slip transform zone, shear is predominantly horizontal and, therefore, vertical rock uplift is weaker (Fig. 3f) despite the highly localized strain rates (Fig. 3b, e)*.*

**Figure 4:**
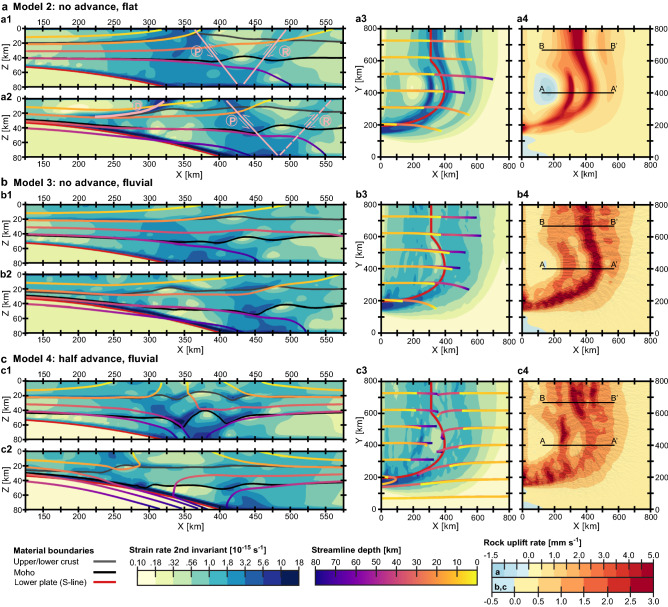
Strain rate (shown in vertical cross-sections and plan view) and rock uplift rates (top view) after 6 Myr﻿ modelling time for (**a**) model 2 (total erosion; no upper plate advance); (**b**) model 3 (fluvial erosion; no upper plate advance); and (**c**) model 4 (fluvial erosion; half upper plate advance). Figure conventions as in Fig. 3. Note that in the models without the upper plate advance component (panels “a” and “b”), the relatively shallow (i.e., originated at 10–20 km depth) particle streamlines show a gradual increase in exhumation of upper and lower crustal material from the left (subduction) side of the model towards its center. In the experiment characterized by fluvial erosion and half upper plate advance (panel “c”), the zones of strong rock uplift at the surface coincide with the three main deformation structures of the model: (1) the orogenic wedge above the cylindrical slab; (2) the indenter-centered decollement; and (3) the transform fault bounding the lateral margin of the subducting plate (see Fig. 3b).

In model 2 (Fig. 4a), in which lateral shortening is accommodated only by subduction (i.e., without upper plate advance), the indenter-centered decollement is shifted in the positive X-direction to the interior of the overriding plate (Fig. 4a2-3). As a result, the distance between peaks in rock uplift in the hanging wall of the decollement and at the continuation of the pro-side shear band into the central part of the model becomes smaller than in model 1, resulting in a narrower basin between these structures (cf. Figs. 4a4 and 3f). Importantly, the pro-shear zone does not weaken in the center of the experimental domain as in model 1 (Fig. 3d–e), but extends continuously from the rear part of the model, where it bounds the lithospheric-scale wedge (Fig. 4a1), towards the lateral boundary of the subducting slab, where it merges directly into the transform fault (Fig. 4a3). Rock uplift at the surface follows this distribution of strain rate and forms two bands of high vertical velocity, one extending along the S-line and the other localized above the indenter apex (cf. Figs. 4a4 and 4a3).

Switching from total erosion to more realistic fluvial erosion (model 3; Fig. 4b) leads to less localized faulting patterns. Strain rates exhibit a broader and more diffuse distribution with values above 5**·**10^−15^ s^−1^ confined to the lower crust and lithospheric mantle (Fig. 4b1-2), except for the transform shear zone in the front part of the model, where localized high deformation occurs at a shallower level (Fig. 4b3). It is noteworthy that despite the lack of concentrated strain rates in the upper crust, strong rock uplift is observed along the entire pro-shear zone in both the rear and middle segments of the model domain. Towards the front part, this trench-parallel structure wraps around the curved lower plate and connects with the area of focused vertical velocities associated with the strike-slip transform (Fig. 4b4). This results in a continuous band of high uplift rates similar to that obtained in the corresponding simulation with flat erosion (model 2, Fig. 4a4). In contrast, the shallowly dipping decollement, which was clearly visible in the central part of the two previous experiments (models 1 and 2), has almost disappeared (Fig. 4b2-3). As a consequence, rock uplift above the indenter is less localized and weaker in amplitude (Fig. 4b4).

Finally, model 4 (Fig. 4c) combines the half upper plate advance (as in model 1; Fig. 3) with fluvial erosion (as in model 3; Fig. 4b). In the rear part of the model, the high rock uplift rates spread over a relatively large area (up to 200 km in the X-direction; Fig. 4c4) related to weakly localized pro- and retro-shear structures in the continental lithosphere overlying the cylindrical segment of the subducting plate (Fig. 4c1). Similar to the corresponding flat erosion case (model 1; see Fig. 3d, f), the continuation of this focused uplift attenuates significantly towards the ellipsoidal indenter in the middle of the model (Fig. 4c4) along with the associated deformation zones (Fig. 4c2). The decollement above the tip of the indenter (Fig. 4c2) is also less pronounced and, in this case, inclined in the pro-direction (i.e., opposite to the retro-decollement in model 1; Fig. 3d). Nevertheless, the deep and nearly vertical exhumation in the hanging wall (see material streamlines in Fig. 4c2) results in a sufficiently well-localized area of concentrated rock uplift centered on the indenter (Fig. 4c4). The third region of rapid rock uplift is located at the lateral boundary of the subducting plate (Fig. 4c4) along the transform fault (Fig. 4c3), which is best expressed in terms of near-surface deformation (compared with the other shear zones), similar to another experiment involving fluvial erosion (model 3; Fig. 4b3). In general, both models with equal partitioning of boundary velocities (models 1 and 4 characterized by half upper plate advance) have similar first-order characteristics in rock uplift rates (cf. Figs. 3f and 4c4). The fluvial erosion scenario (model 4), however, exhibits a less localized and more distributed pattern with a smaller maximum amplitude (~ 3 mm yr^−1^ instead of > 5 mm yr^−1^), yet shows a stronger relative localization of vertical velocities at the surface above the transform fault zone.

### Predicted cooling ages and application to southern Alaska

**Figure 5:**
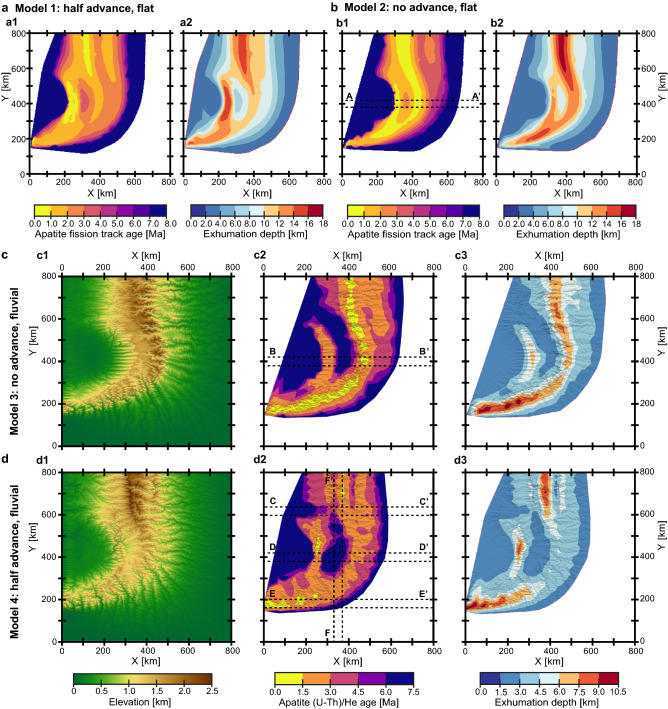
Predicted cooling ages, exhumation depths, and surface topography (shown only for fluvial erosion scenarios) after 6 Myr modelling time for (**a**) model 1 (total erosion; half upper plate advance); (**b**) model 2 (total erosion; no upper plate advance); (**c**) model 3 (fluvial erosion; no upper plate advance); and (**d**) model 4 (fluvial erosion; half upper plate advance). The apatite fission track (AFT) ages (closure temperature T_c_ is 120 ℃) are shown for the total erosion models (panels “a” and “b”) because the rock uplift rates in these experiments are too high to produce much variance in the predicted apatite (U-Th)/He (AHe) ages (T_c_ is 70 ℃). In contrast, the latter are shown for the fluvial erosion cases (panels “c” and “d”), which are characterized by lower vertical velocities at the surface. Since the resulting thermochronological ages are determined not only by the kinematics of the exhumation paths but also by the evolving temperature field within the model, the distributions of predicted cooling ages are complemented by the exhumation depths, which are not affected by thermal processes. Note, however, that because the experiments begin with a laterally uniform temperature structure, the patterns of exhumation depths and predicted cooling ages (as well as rock uplift rates; see Figs. 3, 4) are quite similar. The 40-km swath cross-sections A-A’, B-B’, C–C’, D-D’, E-E', and F-F' marked here by dashed lines, are shown in Fig. 6.

**Figure 6:**
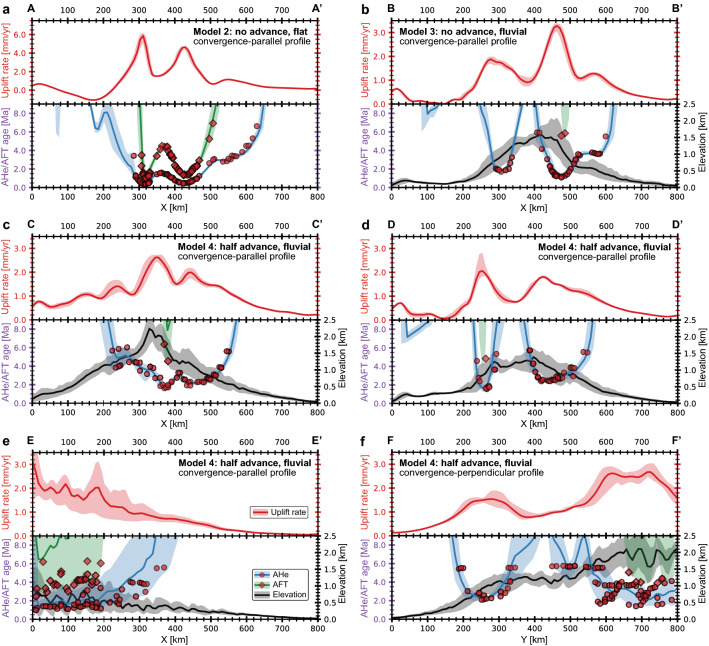
Swath profiles of rock uplift rates (red lines; upper panels), topography, and predicted AHe and AFT ages (black, blue, and green lines; lower panels) for the following experiments after 6 Myr modelling time: (**a**) model 2 (total erosion; no upper plate advance); (**b**) model 3 (fluvial erosion; no upper plate advance); (**c**–**f**) model 4 (fluvial erosion; half upper plate advance). Zones with light colors indicate the variation of the corresponding parameters at 40 km intervals in the profile-perpendicular direction. The red circles (AHe) and diamonds (AFT) indicate the cooling ages extracted from the tracking particles, while the continuous blue (AHe) and green (AFT) lines denote their interpolation (which is also shown in the corresponding map views in Fig. 5). Profiles A-A’, B-B’, C-C’, D-D’, and E-E’ (panels “a” to “e”) are oriented parallel to the convergence, while profile F-F’ (panel “f”) has a subduction-perpendicular orientation. The locations of the profiles are shown by the dashed lines in Fig. 5. Note that the youngest cooling ages do not coincide with the highest topography, but are offset on the slopes of the orogen (panels “b” to “d”). The direct linear relationships between age and altitude are thus applicable only to limited areas, and even an inverse dependence may be reasonable. Model 3 (fluvial erosion; no upper plate advance; panel “b”) is characterized by a skewed topography in which the slope on the retro-side (right) is much steeper than on the pro-side (left), while the corresponding topographic profile is much more symmetrical when boundary velocities are evenly distributed between subducting and overriding plates (model 4; panel “d”).

In these coupled thermo-mechanical/surface processes models, particle tracking allows inspection of the pressure–temperature history of rocks exhumed at the surface and resulting thermochronological cooling ages. According to our results, only the scenarios with half upper plate advance exhibit isolated regions of young apatite (U-Th)/He (AHe) and apatite fission track (AFT) ages above the indenter, whereas a distinctive feature of the models without upper plate advance is a continuous band of young cooling ages along the S-line (cf. panels “a” and “d” with panels “b” and “c” in Fig. 5). Although the general distribution of thermochronological ages in the fluvial erosion scenarios still follows patterns defined by tectonics, the cooling ages vary over shorter length scales, forming localized areas of very young values that are much smaller in size than in the experiments with absolutely efficient (flat) erosion at the surface (cf. panels “c” and “d” with panels “a” and “b” in Fig. 5). In general, the change from total to fluvial erosion reduces the spatial extent of very young ages along the principal (convergence-perpendicular) boundaries of the subducting plate and above the indenter apex. However, fluvial erosion leads to a stronger localization of younger thermochronological ages and higher exhumation depths along the transform fault (Fig. 5). Another important feature of the models with fluvial landscapes is that the youngest AHe ages are found on orogenic slopes, preferably in steep valleys with sufficient upstream drainage area, and therefore do not coincide with the highest topography (Fig. 6).

**Figure 7:**
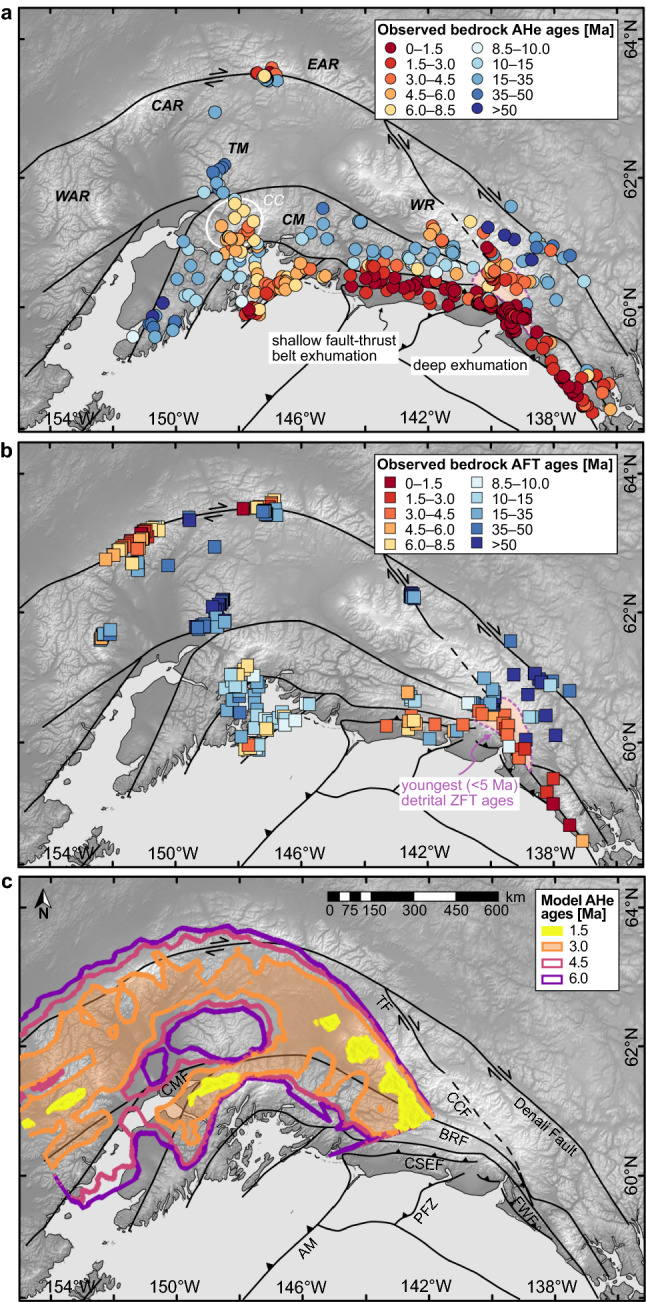
Comparison of observed and modelled spatial distributions of thermochronological ages overlaid on the topography of southeast Alaska and shown with major faults^35,36^. (**a**–**b**) Compilation of measured bedrock AHe (**a**) and AFT (**b**) ages in the southern Alaskan plate corner (see Supplementary Table 1 and references therein). Note that the area of most rapid and deep exhumation (marked by the pinkish polygon with dashed outline) was observed from detrital zircon fission track (ZFT) data from glacial outwash, i.e., from catchment elevations including low, ice-covered elevations where bedrock cannot be sampled directly (see refs.^18,43,52^ for details). Importantly, the very young AHe ages along the area referred to as “shallow fault-thrust belt exhumation” in panel “a” are the result of exhumation along the shallow particle paths in the fold-and-thrust belt^32^ in combination with high precipitation on the coast and therefore should not be considered for comparison with the model data. CAR—Central Alaska Range, CM—Chugach Mountains, EAR—Eastern Alaska Range, TM—Talkeetna Mountains, WAR—Western Alaska Range, WR—Wrangell Mountains. White ellipse: CC—Chugach Core. (**c**) Predicted AHe ages for model 4 (fluvial erosion; half upper plate advance). Note that three observed zones of relatively young thermochronological ages (the Fairweather fault/St. Elias syntaxis, the Chugach Core, and the Alaska Range) find their modelled equivalent in the similar relative positions of isolated regions above the main structures of localized deformation in the crust and mantle of the overriding lithosphere (from east to west: transform fault, decollement, and orogenic wedge; see Fig. 3b). The westernmost zone of young predicted ages (associated with the orogenic wedge) does not exactly match the Alaska Range, where there is insufficient measured data for a good comparison. AM—Aleutian megathrust, BRF—Border Ranges fault, CCF—Connector fault, CMF—Castle Mountain fault, CSEF—Chugach-St. Elias fault, FWF—Fairweather fault, PFZ—Pamplona fault zone, TF—Totschunda fault.

Figure 7 illustrates a comparison of predicted AHe ages for model 4 (fluvial erosion; half upper plate advance) with a compilation of observed ages in southern Alaska. The youngest AHe ages (< 1 Ma; refs.^55,56^) are reported along the Fairweather transform fault including the area of its transition to convergence within the St. Elias syntaxis and connection to the Denali fault to the north. Another observed site of relatively young cooling ages (< 5 Ma; ref.^33^) is located above the subducted part of the Yakutat microplate within the Chugach Core (Fig. 7a). Both zones are reproduced in model 4, which shows younger thermochronological ages focused above the lateral boundary of the subducting slab and the indenter apex (Fig. 7c). Although the tectonic structures associated with these two regions of localized exhumation—dextral strike-slip/transpressional fault and indenter-centered thrust-sense decollement (Fig. 3b)—are expressed to varying degrees in all the experiments presented (Figs. 3–4), their manifestation in concentrated rapid rock uplift within separate zones at the surface is found only in model 4, which includes fluvial erosion (Fig. 4c4). In contrast, the corresponding total erosion experiment (model 1) lacks focused rock uplift along the transform shear zone (Fig. 3f). In another simulation assuming absolutely efficient (flat) erosion at the surface (model 2 without upper plate advance), transform- and decollement-related zones of fast exhumation coalesce at the curved segment of the indenter boundary, where they merge with a wedge-related band that extends continuously from the rear to the middle part of the model box (Fig. 4a4). However, such an elongated zone of localized rock uplift and exhumation has not been observed in nature (Fig. 7a–b). In model 4, the third isolated zone of relatively young predicted ages is associated with the segment of the orogenic wedge that formed over the cylindrical segment of the slab in the rear part of the model domain (Fig. 7c). The young AFT ages in the Central Alaska Range may be a natural analogue (Fig. 7b), although their actual position is somewhat closer to the tip of the subducting part of the Yakutat indenter (Fig. 1) compared to the corresponding area reproduced in the model which is shifted further to the west (Fig. 7c). To improve agreement with the observed relative spatial location of the major structures and to adjust the values of the observed cooling ages, more detailed simulations are required that more accurately account for the actual geometry and composition of the lower oceanic plate, including a potential slab tear towards its eastern edge^40,45^, inherited lateral heterogeneities in the upper continental plate that comprises previously accreted terranes, and additional elements such as oblique convergence and glacial erosion in the surface erosion model (see “Methods” for more details on model limitations).

The influence of surface erosion on the style and distribution of deformation in the crust and lithosphere has long been recognized^63,64^. However, most hybrid geodynamic-geomorphologic modelling studies are still limited to a simplified, purely diffusion implementation of surface processes^65,66^ and/or 2D experiments^67–69^. Some previous studies using 2D model setup are sometimes extended to a 2 + 1 D approach where erosion and sedimentation are defined by the in-plane surface processes model and averaged over the width to account for the cross-sectional thermo-mechanical model. In this approach, the tectonic uplift and subsidence derived from this 2D thermo-mechanical model are then duplicated along the third dimension to provide a horizontal grid of input values for the surface processes model at the next time step^70–73^.

Our results highlight the importance of using a modelling framework that combines true 3D-thermo-mechanical simulations with realistic (including fluvial erosion) landscape evolution in a fully coupled geodynamic-geomorphologic system. This is particularly vital for plate corners and associated syntaxial orogens, where extreme and localized exhumation is, by all appearances, the result of the combined and mutually reinforcing effects of geodynamic and geomorphic processes. We show that the processes operating at the Earth's surface affect not only crustal and lithospheric deformation but also rock uplift patterns. From this perspective, a realistic implementation of fluvial erosion and hillslope diffusion appears to be an important factor in reproducing localized and isolated zones of rapid rock exhumation and observed young thermochronometric ages on the southern margin of Alaska.

## Methods

We produced the numerical simulations presented in this contribution using a fully-coupled numerical approach, that combines state-of-the-art 3D techniques for geodynamic and geomorphologic modelling.

### Thermo-mechanical code

The 3D code DOUAR^2,62^ solves mass and momentum energy conservation equations for an incompressible fluid. Materials can be either purely viscous or frictional visco-plastic. The viscous material deforms according to a thermally-activated creep law. The brittle/plastic regime is formulated in terms of the Mohr–Coulomb failure criterion. The resulting visco-plastic rheologies are implemented via the evaluation of the effective viscosity which is defined as the minimum between the ductile and brittle/plastic components (a “Christmas tree”-like criterion^74,75^). The mechanical equations are fully thermodynamically coupled with the heat conservation equation, which takes into account radiogenic heat sources.

### Landscape evolution code

In the FastScape algorithm^7^, surface processes are described as a combination of fluvial erosion and hillslope diffusion. Fluvial erosion is represented by the stream power law, which predicts the evolution of river channels in a detachment-limited system with no deposition of eroded material. Hillslope diffusion is parameterized as a linear function of slope gradient when the rate of topography change is proportional to the curvature of the topography.

### Coupling geodynamic and geomorphologic modelling techniques

At each time step of the geodynamic model (DOUAR), the geometry of the upper surface is modified by surface processes as calculated by the landscape evolution model (FastScape), which considers not only the implemented erosion and diffusion mechanisms but also the vertical (tectonic uplift/subsidence) and horizontal velocities derived from DOUAR. With this approach, we ensure a complete two-way coupling of the thermo-mechanical and the surface erosion components of the geodynamic-geomorphologic modelling.

More detailed information on the algorithms for geodynamic and geomorphologic modelling (including the governing equations and the rheological model) and the approach to coupling them can be found in the Supplementary Methods.

### Model setup

DOUAR model encompasses an area with a horizontal extent of 800 km × 800 km and a vertical thickness of 80 km. The spatial resolution is 6.25 and 1.54 km in the horizontal and vertical directions, respectively.

The laterally homogeneous overriding plate is modelled as rheologically stratified continental lithosphere with a vertical alternation of brittle and ductile rock behaviors (Supplementary Fig. 1a). The temperature-dependent visco-plastic rheology adopts the ductile flow laws of wet granite for the upper crust (20-km-thick) and dry diabase for the lower crust (20-km-thick) (both ref.^76^). The 40-km-thick lithospheric mantle follows the creep law of olivine aggregates^77,78^. In the upper and lower crust, the brittle strength undergoes a linear reduction with increasing accumulated strain (linear strain softening^79^). The initial temperature distribution (Supplementary Fig. 1b) corresponds to the nonlinear steady-state geotherm defined by boundary temperatures of 0 °C (Z = 0 km) and 930 °C (Z = 80 km) and radiogenic heat production in the crustal layers, which results in a Moho temperature of 600 °C and a heat flux of 70 mW m^−2^ at the surface. The thermal and rheological parameters used in our study are listed in Supplementary Table S2.

The downgoing plate is asymmetric. Its rear portion (600 km < Y < 800 km) consists of a cylindrical slab with a minimum depth (Z = 50 km) at X = 0 km, gradually increasing towards the S-line (Z = 80 km at X = 320 km). The adjacent segment (200 km < Y < 600 km) is represented by an ellipsoidal indenter with a shallower minimum depth (Z = 30 km) and a more distant termination (at X = 400 km) in its central section (Y = 400 km). This configuration provides a lower dip angle of the indenter bulge compared to the neighboring cylindrical slab. The indenter is more tightly curved in the Y-direction (200 km radius), resulting in the absence of the subducting plate in the front part of the model (0 km < Y < 200 km). The mechanical coupling with the overriding plate is mitigated by a 3–4 km thin and weak interface layer, which ensures a balance between traction from the downward moving subducting plate and the ability of upper plate material to shear away from the interface. In contrast to the overriding continental lithosphere, constant viscosity values are assumed for the entire subducting plate and the interface layer (10^25^ Pa s and 10^21^ Pa s, respectively). We refer the reader to our previous studies for a detailed analysis of the viscosity of the interface layer^24^ and the rheological structure of the overriding continental plate^16^.

Uniform and time-independent velocities are applied to the left (X = 0 km) and right (X = 800 km) sides of the model domain in opposite directions parallel to the X-axis. The overall rate of shortening remains the same in all experiments (30 mm yr^-1^), while the ratio between upper plate advance and lower plate subduction (i.e., the velocities applied to the left and right model boundaries, respectively) is a variable parameter (half or no upper plate advance component). Note that the front part (0 km < Y < 200 km) of the left boundary (X = 0 km) is set to the same velocity as the right side (X = 800 km) since it belongs to the overriding plate. To avoid a sharp contrast of velocities at the left boundary (X = 0 km), a 40 km wide linear transition is applied along the vertical zone around Y = 200 km. The downgoing plate is characterized by rotational motion, with horizontal inflow at the left boundary (X = 0 km; 200 km < Y < 800 km) compensated by vertical outflow through the lower boundary (Z = 80 km) within the slab area. At the front (Y = 0 km) and back (Y = 800 km) sides, free slip velocity boundary conditions are imposed. The resulting distribution of velocities within the model domain implies a transform motion along the lateral boundary of the subducting slab at Y = 200 km, which transitions to a perpendicularly oriented convergence along its principal boundary at X = 320–400 km.

It is worth noting that we intentionally simplify our model by dictating that the downgoing plate (including the indenter) remains a nearly undeformed mass and retains its original configuration throughout the entire model history. With this approach, we can rule out mass transfer between the subducting and overriding plates and thus identify the isolated effect of a strong subducting indenter on the deformation pattern in the rheologically layered continental lithosphere. We assume that the general findings on the dominant influences on upper plate tectonics are valid for the uppermost segment of the lithosphere-mantle system (0–80 km), since most of the internal deformation in the subducting slab normally occurs at much greater depths^80^.

For the non-flat surface erosion experiments, FastScape calculations were performed on a regular grid with a resolution of 0.78 km and a uniform distribution of fluvial erosion efficiency (8**·**10^–6^ m^1-2* m*^ yr^−1^ with an area exponent *m* of 0.4) and hillslope diffusion coefficient (4 m^2^ yr^−1^).

To account for the flexural response to compressional tectonic forces and surface erosional unloading, all surfaces in the DOUAR 3D grid are subjected to an isostatic adjustment calculated assuming an effective elastic thickness^81^ of 30 km.

### Limitations of the model and future perspectives﻿

Although the setup of our experiments captures the main features of the subducting plate configuration, the laterally homogeneous overriding plate does not encompass the various elements of the crustal and lithospheric structure of the studied area^38,46^. The contribution of lithospheric buoyancy forces to internal lithospheric stresses and deformations is known from previous global^82–84^ and regional^85–87^ numerical geodynamic models and, therefore, warrants thorough further investigation in upcoming studies with the application of cutting-edge technologies such as coupled geodynamic-geomorphologic modelling. Moreover, lateral differences in subducting plate properties may also be important: for example, oceanic plateaus are known to have a thicker crust^88^, resulting in higher lithospheric buoyancy relative to the surrounding oceanic lithosphere^89^. In addition, the oblique components of convergence need to be considered in order to account for the strike-slip tectonics of trench-parallel structures such as the Denali fault.

The uniform distribution of fluvial erosion efficiency over the entire modelling surface should also be improved in future models, for example by incorporating a physically-based orographic precipitation model^90^, which could provide additional insight into the feedbacks between tectonic uplift and surface erosion. The glacial erosion components, including snow avalanching and iceberg calving^91,92^ are to be integrated into the geomorphic part of the model as well.

Finally, it is noteworthy that the configuration of our model is applicable to plate corners where the downgoing plate subducts and collides along a concave boundary relative to the overriding plate, as in southern Alaska^18,33,34,43^, while a convex shape of the convergent boundary^93^, such as in the Southeast Caribbean plate corner^94^, remains outside the scope of our study.

## Supplementary Information

Supplementary Information 1.

Supplementary Information 2.

Supplementary Information 3.

Supplementary Information 4.

## Data Availability

The data supporting the findings of this study are available within the article and its Supplementary Information files.
