# Supplementary material for: 3D geodynamic-geomorphologic modelling of deformation and exhumation at curved plate boundaries: Implications for the southern Alaskan plate corner
Source: Sci Rep. 2022 Aug 22;12:14260. doi: 10.1038/s41598-022-17644-8 (PMC9395393; doi:10.1038/s41598-022-17644-8)
Supplement: Supplementary file 1 — Supplementary Information 1. [file 41598_2022_17644_MOESM1_ESM.pdf]

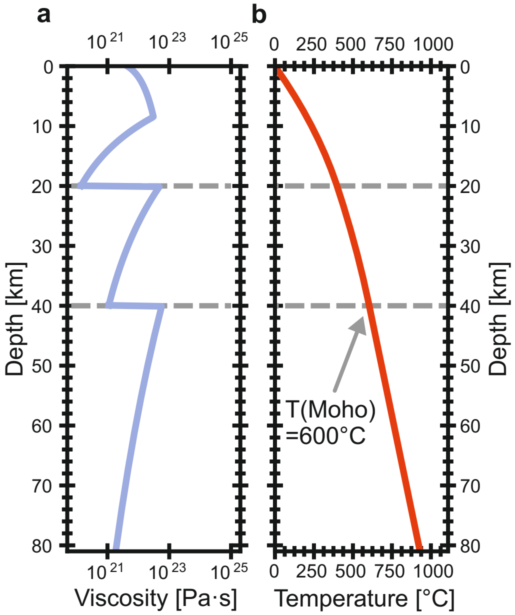

Supplementary Figure 1. (**a**) The effective viscosity in the overriding continental plate defined by a Christmas tree-like criterion (Ranalli, 1995; Burov, 2011), assuming the ductile flow laws of wet granite and dry diabase (Carter & Tsenn, 1987) for the upper and lower crust and the creep law of olivine aggregates (Hirth & Kohlstedt, 2003; Jadamec & Billen, 2012) for the mantle. The presented vertical profile was calculated for the initial temperature distribution (see panel "**b**") and a constant strain rate of 10^{-15} s^{-1}.

(**b**) Vertical profile of initial temperature. Nonlinear steady-state geotherm is defined by boundary temperatures, taking into account radiogenic heat production in crustal layers. The rheological and thermal parameters are given in Supplementary Table 2.
