# Supplementary material for: 3D geodynamic-geomorphologic modelling of deformation and exhumation at curved plate boundaries: Implications for the southern Alaskan plate corner
Source: Sci Rep. 2022 Aug 22;12:14260. doi: 10.1038/s41598-022-17644-8 (PMC9395393; doi:10.1038/s41598-022-17644-8)
Supplement: Supplementary file 2 — Supplementary Information 2. [file 41598_2022_17644_MOESM2_ESM.pdf]

Supplementary Methods for

3D geodynamic-geomorphologic modelling of deformation and exhumation at curved plate boundaries: Implications for the southern Alaskan plate corner

Alexander Koptev[1,2], Matthias Nettesheim[1], Sarah Falkowski[1], Todd A. Ehlers[1]

[1]Department of Geosciences, University of Tübingen, Tübingen, Germany

[2]GFZ German Research Centre for Geosciences, Potsdam, Germany

1. Geodynamic thermo-mechanical modelling

1.1. Governing equations

The 3D thermo-mechanical numerical code DOUAR (Braun et al., 2008; Thieulot et al., 2008) was developed to solve the conservation of mass, momentum and energy for an incompressible fluid. DOUAR solves Stokes flow equations consisting of momentum conservation (equation 1) and mass conservation (equation 2):

$$\nabla \cdot \ \mu\,(\nabla u + \nabla u^T) - \nabla P = \rho g; \qquad (1)$$

$$\nabla \cdot u = 0, \qquad (2)$$

where μ is effective viscosity, u is velocity field, P is pressure, ρ is density, and g is acceleration due to gravity.

Pressure can be eliminated from these equations by introducing a so-called penalty or compressibility factor, λ:

$$P = -\lambda \nabla \cdot u. \qquad (3)$$

The compressibility factor is usually eight orders of magnitude larger than the shear viscosity to ensure nearly incompressible behavior of the flow.

The mechanical equations are coupled with the heat conservation equation:

$$\rho c_p \left(\frac{\partial T}{\partial t} + u \cdot \ \nabla T\right) = \nabla \cdot k \nabla T + \rho H_r, \qquad (4)$$

where T is temperature, c_p is heat capacity, k is thermal conductivity, and H_r is radiogenic heat production (see Supplementary Table 2).

1.2. Rheological model

The material deforms according to a thermally-activated creep law:

$$\mu = B\ \dot{\varepsilon}^{1/n-1} e^{Q/nRT}, \qquad (5)$$

where μ is viscosity, $\dot{\varepsilon} = \sqrt{1/2\,\dot{\varepsilon}_{ij}\dot{\varepsilon}_{ij}}$ is second invariant of the strain rate tensor, and B is pre-exponential factor, Q is activation energy, n is power-law exponent, and R is gas constant (Supplementary Table 2).

When plasticity is activated, material deformation is determined by the Mohr-Coulomb failure criterion:

$$\tau = C - \sigma_n tan\varphi, \qquad (6)$$

where τ is shear stress, σ_n is normal stress, C is cohesion, and φ is material friction angle (Supplementary Table 2). The friction angle decreases with increasing total strain (linear strain softening).

The visco-plastic rheology is assigned to the model using a Christmas tree-like criterion, where the rheological behavior is defined by the minimum differential stress (or viscosity) reached between ductile and brittle/plastic fields (Ranalli, 1995; Burov, 2011).

2. Geomorphologic modelling

At each time step in the geodynamic model, the geometry of the upper surface is subjected to surface processes modelled by the FastScape code (Braun & Willett, 2013), which incorporates the effects of fluvial erosion and hillslope diffusion.

In the FastScape algorithm, fluvial erosion is represented by the stream power law, which is commonly used to predict the evolution of river channels in a detachment-limited system (i.e., bedrock incision by rivers in steep mountainous landscapes):

$$\frac{\partial h}{\partial t} = U + v \cdot \nabla h - K \cdot (P \cdot A)^m \cdot S^n, \qquad (7)$$

where h is topographic height, t is time, U and v are tectonic uplift/subsidence and lateral velocity (both derived from the geodynamic model), K is erodibility coefficient, P is precipitation, A is drainage area, S is local slope, and m and n are positive exponents.

For hillslope diffusion, the rate of topography change is proportional to the curvature of the topography. Combining the two processes gives the following equation for landscape evolution:

$$\frac{\partial h}{\partial t} = U + v \cdot \nabla h - K \cdot (P \cdot A)^m \cdot S^n + K_d \nabla^2 h, \quad (8)$$

where K_d is transport coefficient or diffusivity.

The values of the surface processes parameters can be found in the main text of the manuscript.

3. Coupling of DOUAR and FastScape

As mentioned above, surface erosion simulations were performed at each time step of the geodynamic model to ensure full bidirectional coupling of the thermo-mechanical processes modelled in DOUAR and the landscape evolution modelled in FastScape. Thus, each iteration of the coupled DOUAR/FastScape model executes the following four substeps: 1) the DOUAR velocity field is computed from the solution of Stokes flow equations (see equations 1-2 in Section 1.1); 2) the velocity field is interpolated onto a regular mesh representing the model surface. This surface mesh has a higher horizontal resolution (0.78 km instead of 6.25 km in DOUAR) to allow more accurate modelling of landscape evolution; 3) the surface topography is modified in FastScape according to the velocities derived from DOUAR and the implemented erosion and diffusion mechanisms (see equation 8 in Section 2); 4) the resulting topography is transferred back to DOUAR where it is used as the initial condition for the next iteration.
