# Supplementary material for: 3D geodynamic-geomorphologic modelling of deformation and exhumation at curved plate boundaries: Implications for the southern Alaskan plate corner
Source: Sci Rep. 2022 Aug 22;12:14260. doi: 10.1038/s41598-022-17644-8 (PMC9395393; doi:10.1038/s41598-022-17644-8)
Supplement: Supplementary file 4 — Supplementary Information 4. [file 41598_2022_17644_MOESM4_ESM.pdf]

Supplementary Table 2. Density and rheological and thermal parameters for the overriding continental plate.

Material	*Density* ρ $[\mathrm{kg \cdot m^{-3}}]$	*Rheological parameters*							*Thermal parameters*	
		Ductile				*Brittle/plastic*			*Thermal diffusivity* $\alpha = \frac{k}{\rho c_p}$ $[10^{-6} \times \mathrm{m^2 \cdot s^{-1}}]$	*Heat production* H_r $[\mu W \cdot \mathrm{kg^{-1} \cdot m^{-3}}]$
		Flow law	*Activation energy* Q $[\mathrm{kJ \cdot mol^{-1}}]$	*Power-law exponent* n [ ]	*Pre-exponential factor* B $[\mathrm{Pa \cdot s^{1/n}}]$	*Cohesion* C [MPa]	*Friction angle* φ [°]	*Strain weakening interval* [ ] –> [ ]		
**Upper crust**	2750	Wet granite[a]	140.6	1.9	4.43×10^7	10	15 –> 5	0.05 –> 0.55	1.0	1.8
**Lower crust**	2900	Dry diabase[a]	276.0	3.05	1.24×10^6	10	15 –> 5	0.05 –> 0.55	1.0	0.6
**Lithospheric mantle**	3300	Olivine aggregates[b]	324.3	3.5	1.21×10^7	2	10	–	1.0	0

[a] Carter & Tsenn (1987)

[b] Hirth & Kohlstedt (2003); Jadamec & Billen (2012)
